# Screening of Living Kidney Donors for Genetic Diseases: Commentary

**DOI:** 10.34067/KID.0000000000000470

**Published:** 2024-05-15

**Authors:** Robert W. Steiner

**Affiliations:** UCSD Center for Transplantation and Division of Nephrology, University of California at San Diego School of Medicine, San Diego, California

**Keywords:** genetic renal disease, kidney donation, renal transplantation, risk factors

A genetic basis has been suggested for increased ESKD risks in living kidney donors who are closely related to their recipients, in Black living donors compared with non-Black donors, and in living donors compared with matched nondonor controls.^1–3^ This issue of *Kidney360* includes two authoritative perspectives on testing for genetic risks in such donor candidates.^4,5^ Both submissions recommend focused donor testing when a monogenetic disease is identified in an affected relative, which is usually the recipient. Neither recommends unfocused screening panels nor whole genome or exome sequencing, citing an extensive and sometimes conflicting literature, predictive uncertainties, the likelihood of false positives, insurance difficulties, and employment risks. One submission identifies ten difficulties of genetic screening per se, seven of which are present in both universal and focused approaches.^5^ Both submissions recognize the importance of appropriate counseling with genetic testing, which often may not be achieved. Both submissions endorse a significant but largely unrecognized prevalence of genetically caused or influenced ESKD. One submission advocates that APOL1 (apolipoprotein L1) testing be offered to candidates with African ancestry, but not required.^4^ The other advocates that donor acceptance of genetic testing should never be a prerequisite for donor approval and notes that increased relative donor risks are tempered by low absolute risks.^5^

A recent review by the Living Donor Community of Practice Genetics Workgroup—which included all three currently contributing authors—provides seven hypothetical vignettes to illustrate appropriate genetic testing of donor candidates.^6^ The first features a 34-year-old donor with a 75-year-old grandfather with PKD1. Three more vignettes feature siblings or parents aged 36, 27, and 45 years, respectively, with recipients whose kidney diseases began in childhood. A fifth involves a 65-year-old woman whose daughter's FSGS began at age 34 years, with CKD in several male relatives with an Alport's variant, which was also present in the daughter but not in the donor. In all these vignettes, the donors had normal medical evaluations, the genetics were relatively well understood, and all were allowed donation after focused testing did not demonstrate a shared, recognized genetic cause of kidney disease. The remaining two vignettes involved APOL1 in donors with African ancestry. In one, donation was allowed after testing was refused by both a medically normal 30-year-old and his 56-year-old mother with hypertensive ESKD. In the second, a medically normal 58-year-old with two high-risk (G1/G1) alleles was allowed donation to a brother with classic diabetic nephropathy and neuropathy. All parties agreed that risk was acceptable, as quantified by an online algorithm and doubled because of APOL1 results. This review also recognized potentially strong disagreements with these decisions, possible inexpert or self-interested donor counseling, the need to respect donor autonomy, and the potential for donor misfortune to erode public confidence in living donation. The importance of ethical treatment of marginalized groups was also noted.

Centers that do not perform genetic testing may learn from these examples. Genetic ESKD can be misattributed to hypertension, which by itself almost never causes ESKD. It should be suspected in atypical diabetic nephropathy, with young recipients, and with kidney diseases in multiple family members. Because genetic diseases often begin early in life, routine screening may rule out older affected candidates. Older medically normal Black donors may have low APOL1-associated risks.^3^ Genetic testing will also make donor counseling more complicated. Currently counseling conveys exceedingly low risks, which are easy to understand, codify, and accept. Genetic counseling includes nonmedical issues, familial implications, and possibly higher and more complicated risks. Shared decision making requires centers to first formulate well-considered, defensible options, and donor understanding should be well documented.^7^

**Figure 1:**
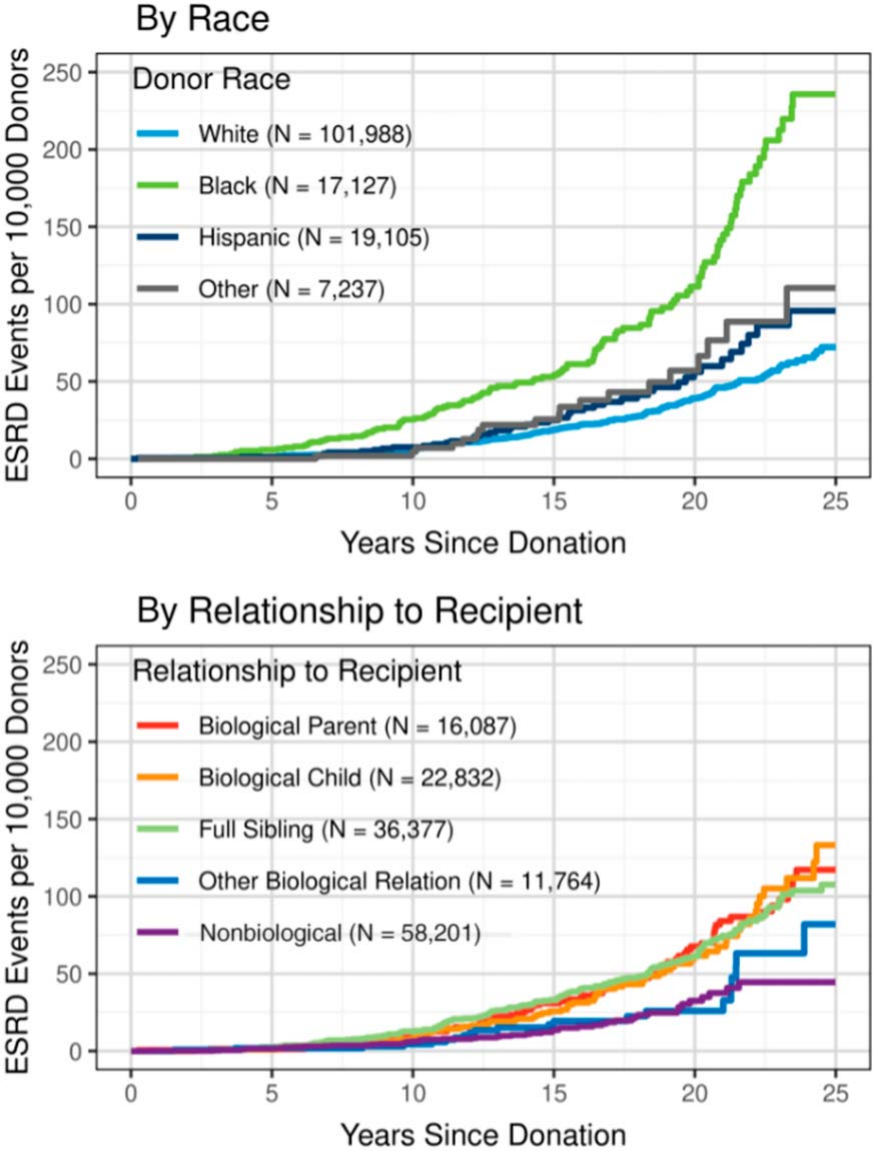
**Increase in ESKD in kidney donors who are close relatives and in Black donors.** From ref. 1, with permission.

All three *Kidney360* authors offer only a qualified, conditional commitment to genetic testing in its current state. By comparison, we would not give donors freedom to decline aspects of standard testing. For example, evaluations for hematuria are not optional, although it is a weak predictor of disease. At some point, the imperfections of genetic testing may not outweigh its unique benefit. As one submission suggests,^5^ time-honored protocols allow donation to candidates without clinical abnormalities, but genetic testing further stratifies risk in medically normal individuals. Indeed, classic teachings have overestimated the ability of current selection protocols to predict truly new-onset postdonation kidney diseases. No biologic mechanism explains how being medically normal at donation could detect genetic risk or prevent a panoply of nongenetic future diseases,^8^ and outcome data do not justify this belief. To be sure, 10-year donor ESKD rates are exceedingly low, but they are just as low in entirely unselected young adults in the general population.^8,9^ Both cohorts are largely normal initially, and in both, kidney diseases will begin, accumulate, and typically progress slowly. As per Figure 1, undetected genetic diseases make large contributions to rates of ESRD accumulation in donors, which appear to be exceeding rates of accumulation in the general population through 30 years.^8,9^ Young, long-lived donors will have substantial—although distant—cumulative lifetime ESKD risks and proportionately greater risks of advanced CKD.^8–10^ Because of their disproportionate contribution to cohort risk, closely related donors and Black donors may have ESKD risks that exceed the cohort average (Figure 1, ref. 1). Currently, we do not know what to expect on the individual level, which may in part be addressed with genetic testing. Black donor risk is especially concerning. We currently allow Black donors to take more risk than others, which is more than bad optics. We should either allow higher risks for non-Black donors or reduce allowable Black risk. While not completely predictive, APOL1 status is a risk factor which would help define individual risk in Black candidates.^10^

In summary, our maturing living kidney donor databases have documented important, unforeseen risks that genetic testing could in principle detect. The reasoned judgment of experts to not endorse fully genetic testing in its current state—even in limited contexts—must be respected. It will remain critical to clarify genetic risks so donors and centers can deal with them more confidently. The *Kidney360* submissions suggest promise in this complicated, ethically charged area.
